# Nasogastric tube feeding versus assisted hand feeding in-home healthcare older adults with severe dementia in Taiwan: a prognosis comparison

**DOI:** 10.1186/s12877-020-1464-9

**Published:** 2020-02-14

**Authors:** Hsiao-Hui Chou, Meng-Ting Tsou, Lee-Ching Hwang

**Affiliations:** 1 Department of Family Medicine, Mackay Memorial Hospital, Taipei, Taiwan; 2 Department of Medicine, Mackay Medical College, Taipei, Taiwan

**Keywords:** Dementia, Nasogastric tube, Tube feeding, Hand feeding, Older adults, Home healthcare, Pneumonia

## Abstract

**Background:**

All individuals with severe dementia should be offered careful hand feeding. However, under certain circumstances, people with severe dementia have a feeding tube placed. In Taiwan, tube feeding rate in demented older home care residents is increasing; however, the benefits of tube feeding in this population remain unknown. We compared the clinical prognosis and mortality of older patients with severe dementia receiving nasogastric tube feeding (NGF) or assisted hand feeding (AHF).

**Methods:**

Data from the in-home healthcare system between January 1 and December 31, 2017 were analyzed to identify 169 participants over 60 years of age in this retrospective longitudinal study. All subjects with severe dementia and complete functional dependence suffered from difficulty in oral intake and required either AHF or NGF. Data were collected from both groups to analyze pneumonia, hospitalization, and mortality rates.

**Results:**

A total of 169 subjects (56 males and 113 females, aged 85.9 ± 7.5 years) were analyzed. 39 required AHF and 130 NGF. All subjects were bedridden; 129 (76%) showed Barthel index < 10. Pneumonia risk was higher in the NGF group (48%) than in the AHF group (26%, *p* = 0.015). After adjusting for multiple factors in the regression model, the risk of pneumonia was not significantly higher in the NGF group compared with the AHF group. One-year mortality rates in the AHF and NGF groups were 8 and 15%, respectively, and no significant difference was observed after adjustment with logistic regression (aOR = 2.38; 95% CI, 0.58–9.70). There were no significant differences in hospitalization rate and duration.

**Conclusions:**

For older patients with dementia requiring in-home healthcare, NGF is not associated with a significantly lower risk of pneumonia than AHF. Additionally, neither mortality nor hospitalization rates decreased with NGF. On the contrary, a nonsignificant trend of increased risk of pneumonia was observed in NGF group. Therefore, the benefits of NGF are debatable in older patients with severe dementia requiring in-home healthcare. Continued careful hand feeding could be an alternative to NG feeding in patients with severe dementia. Furthermore, large-scale studies on in-home healthcare would be required to support these results.

## Background

Dementia is a neurodegenerative disease characterized by a progressive, irreversible, and long-term course. Its recent high prevalence rate in Western and Asian countries has a significant impact on modern society [1]. A recent study showed that in Taiwan, the prevalence of dementia was 26.8% in residential houses and 61.8% in assisted living facilities [2]. Therefore, the National Health Insurance in Taiwan has been reimbursing costs of palliative and home care systems for severe dementia. However, owing to the lack of understanding in recognizing progressive and irreversible disease courses, palliative care for severe dementia is not generally accepted as a standard treatment. Life-sustaining treatments in severe dementia remain a major issue. Palliative care decreases life-sustaining interventions, except for tube feeding [3].

In a Chinese-based culture, eating disorders are considered problematic. Therefore, most healthcare proxies choose nasogastric tube feeding. As a result, the rate of NGF has increased over time in Taiwan [4]. A study in a nursing home reported a tube feeding rate of 29.2%, among which 99% was NGF [5].

Nevertheless, the benefits of tube feeding in older individuals with severe dementia and eating difficulties are controversial. Some studies have showed that tube feeding could increase the survival rate and improve nutritional status [6] [7]. Meanwhile, more retrospective observational studies and reviews have indicated that tube feeding offers no benefits in terms of survival, pneumonia risk, pressure ulcers, and nutritional status while increasing physical suffering and impairing the quality of life [8] [9].

Additionally, guidelines published by several professional societies have shown no benefits of NGF and suggest that it should be avoided in patients with severe dementia [10–12]. The first government-sponsored guidebook for palliative care in dementia was published in Taiwan in October 2016. The guidebook supports assisted hand feeding (AHF) in severe dementia and describes numerous feeding skills [13].

Previous studies conducted thus far have collected data from hospitals, nursing homes, or geriatric institutions. However, there are hardly any studies of in-home healthcare systems. Patients with advanced dementia are under-recognized for their high mortality rate and are often treated at in-home healthcare systems with life-sustaining interventions. A better understanding of the real-world scenario of in-home care may provide more information to improve severe dementia care. We conducted a 12-month retrospective longitudinal study of 169 older adults with severe dementia in home healthcare system to compare the prognosis between AHF and NGF.

## Methods

### Study design and data source

Th is was a retrospective observational study. Data were collected from the in-home healthcare system in Mackay Memorial Medical Center, Taiwan. Our government started developing the in-home healthcare system in 1987. With increased aging population, the Ministry of Health and Welfare decided to include in-home healthcare system in the National Health Insurance. Currently, the home healthcare system is well developed and can provide good professional healthcare at home. Barthel index was used as an initial screening tool for home care system registration. Specifically, the Barthel index uses scales to measure 10 basic items of self-care and physical dependency. Enrollment requirement, a Barthel index of < 60, which is associated with assisted independence (normal score 100) [14]. Once the residents were enrolled in the home care system, each individual underwent baseline assessments. Owing to the high demand of home care quality, numerous physical examinations, questionnaire surveys, and psychosocial status assessments were carefully performed. The database contained the following regular home care records: patient age, sex, functional status, care provided by caregivers or family members, feeding status, stool or urinary incontinence, and pressure sores. The Eastern Cooperative Oncology Group (ECOG) performance status was measured on a scale from zero to five; the higher the scores, worse was the performance status. Norton scale [15] was used to predict pressure-induced injuries. Specifically, a score of ≤14 indicated a high risk of pressure ulcer development. Mini Nutritional Assessment (MNA) was applied to assess global nutrition and various body measurements. The time-saving Mini Nutritional Assessment–Short Form (MNA-SF) comprises six questions obtained from the full MNA and has been validated to show good sensitivity [16]. Body mass index (BMI) is a valid predictor of adiposity and can be a practical tool for obesity or underweight assessment. Laboratory data [i.e., serum albumin, hemoglobin (Hb), white blood cell (WBC), creatinine, and electrolyte levels] were collected at baseline and during follow-up. Additionally, underlying comorbidities and hospitalizations were recorded. Home care doctors and nurses visited registered patients from 2 weeks to 3 months and annually, according to patients’ medical conditions.

### Selection of the study subjects

Subjects were selected from the home care registered system. In 2017, 386 individuals with documented cognitive impairment due to dementia were cared for in a home care system. Inclusion criteria consisted of: documented dementia in chart reviews; scores of ≥7A by the Functional Assessment Staging Test (FAST) [17] as evaluated by home-visiting physicians; fully dependent for functional status, as rated by ECOG 4; eating difficulties, as evaluated by home healthcare nurses and physicians; and age ≥ 60 years. Exclusion criteria were as follows: self-oral intake without any assistance; absence of outpatient or inpatient medical records in 2017; and missing information (i.e., MNA-SF, BMI, serum albumin, Hb, or WBC). Finally, a total of 169 subjects with severe dementia, eating difficulty and complete bedridden status (ECOG 4) were enrolled in this study.

In the AHF group, the patients were unable to eat by themselves and required to be fed by another person. In the NGF group, the enrolled subjects were under long-term NG tube placement and regular tube changing in the home healthcare system.

Dementia was classified into 3 subgroups using the diagnosis obtained from medical records. Dementia types included Alzheimer’s disease, vascular dementia, and other causes. Underlying comorbidities were defined on the basis of inpatient or outpatient medical records (ICD-10-CM).

### Outcome measurements

Outcomes were pneumonia risk, hospitalization rate, duration of hospitalization and one-year mortality in the AHF and NGF groups. The diagnosis of pneumonia was cautiously made by reviewing the charts of the patients: [1] admission records, [2] discharge reviews, and [3] diagnosis with pneumonia or aspiration pneumonia. We did not include subjects with pneumonia diagnosis in outpatient records. Second, events of hospitalization were confirmed by admission and discharge notes. Finally, one-year mortality rates were evaluated from discharge note reviews, and home healthcare system records.

### Statistical analysis

IBM SPSS Statistics 25.0 was used for the statistical analysis.

Chi-square test and Fisher’s exact test were performed for categorical variables; Student’s *t*-test for continuous variables were used to compare the differences between the AHF and NGF groups. Significant differences in pneumonia risk, hospitalization rate and duration, and one-year mortality rates between AHF and NGF groups were assessed. Binary logistic regression was performed with adjustments for sex, age, feeding status, Barthel index, pressure sores, Norton scale, serum albumin, Hb, and WBC levels.

A two-tailed *p* value < 0.05 was considered significant.

## Results

### Characteristics of the subjects

Among the 2017 home care residents, 386 patients presented with medically documented dementia. Finally, 169 individuals who met the eligibility criteria were enrolled in the study. There were 39 (23%) and 130 (77%) in the AHF and NGF groups.

Characteristics of AHF and NGF participants with advanced dementia enrolled in the study are summarized in Table 1. Briefly, mean patient age was 85.9 years old and 33% were male. The Barthel index were significantly different between the AHF and NGF groups (*p* < 0.001). Regarding the nutritional status, BMI was significantly higher in the AHF group than in the NGF group (22.7 ± 3.3 and 21.4 ± 3.6, respectively, *p* = 0.042). Moreover, the mean serum albumin level was significantly lower in the NGF group than in the AHF group (3.7 ± 0.6 vs. 4.0 ± 0.6; *p* = 0.045).

**Table 1:** Characteristics of subjects with severe dementia in AHF and NGF

	Total	AHF	NGF	*p*
Numbers no. (%)	169	39 (23)	130 (77)	
Sex (Male) no. (%)	56 (33)	10 (26)	46 (35)	0.257
Age (years, mean ± SD)	85.9 ± 7.5	86.7 ± 6.8	85.7 ± 7.7	0.458
Barthel index (< 10) no. (%)	129 (76)	21 (54)	108 (83)	<.001
Caregiver no. (%)	95 (56)	19 (49)	76 (59)	0.282
Diagnosis				
Alzheimer’s disease no. (%)	24 (14)	3 (8)	21 (16)	
Vascular dementia no. (%)	32 (19)	9 (23)	23 (18)	
Others no. (%)	113 (67)	27 (69)	86 (66)	
Pressure sores no. (%)	27 (16)	6 (15)	21 (16)	0.908
BMI (kg/m^2^, mean ± SD)	21.7 ± 3.6	22.7 ± 3.3	21.4 ± 3.6	0.042
MNA-SF (mean ± SD)	9.3 ± 2.7	9.9 ± 2.5	9.1 ± 2.7	0.103
Norton scale (mean ± SD)	10.1 ± 2.1	10.7 ± 3.1	10.0 ± 1.6	0.161
Serum albumin (g/dL, mean ± SD)	3.8 ± 0.6	4.0 ± 0.6	3.7 ± 0.6	0.045
Hb (g/dL, mean ± SD)	11.0 ± 1.7	11.0 ± 1.9	11.0 ± 1.6	0.942
WBC (10^3^/μL, mean ± SD)	10.4 ± 3.9	9.8 ± 3.1	10.6 ± 4.0	0.296

Abbreviations: *AHF* assisted hand feeding, *NGF* nasogastric tube feeding, *MNA-SF* Mini Nutritional Assessment–Short Form, *BMI* Body Mass Index

*p* < 0.05 for significant difference

### Pneumonia risk

Throughout the 12 months follow-up period, the risk of pneumonia was significantly higher in the NGF group than in the AHF group (48 and 26%, respectively, *p* = 0.015) (Table 2). Multiple variables were examined to perform pneumonia risk assessment (i.e., male sex, pressure sores, feeding status and serum albumin level; Table 2). Regression model adjusted for age, sex, feeding status, pressure sores, Barthel index and serum albumin levels showed that NGF was not associated with decreasing pneumonia risk compared with AHF (aOR = 2.20; 95% CI, 0.92–5.30; Table 3).

**Table 2:** Variables associated with pneumonia, hospitalization and one-year mortality rates

	Pneumonia			Hospitalization			Mortality		
Variables	Yes	No	*P*	Yes	No	*P*	Yes	No	*P*
Total no. (%)	72 (43)	97 (57)		107 (63)	62 (37)		22 (13)	147 (87)	
Age (years, mean ± SD)	85.9 ± 8.2	85.9 ± 7.0	0.990	85.9 ± 7.6	85.9 ± 7.5	0.974	88.4 ± 6.0	85.6 ± 7.7	0.105
Sex (M) no. (%)	31 (43)	25 (26)	0.018	37 (35)	19 (31)	0.601	4 (18)	52 (35)	0.146
Feeding (NGF) no. (%)	62 (86)	68 (70)	0.015	86 (80)	44 (71)	0.162	19 (86)	111 (76)	0.415
Feeding (AHF) no. (%)	10 (14)	29 (30)	0.015	21 (20)	18 (29)	0.162	3 (14)	36 (25)	0.415
Barthel index (< 10) no. %	59 (82)	70 (72)	0.139	79 (74)	50 (81)	0.315	19 (86)	110 (75)	0.292
Pressure sore no. %	5 (7)	22 (23)	0.006	10 (9)	17 (27)	0.002	3 (14)	24 (16)	1.000
BMI (kg/m^2^, mean ± SD)	21.5 ± 3.1	21.8 ± 3.9	0.495	21.8 ± 3.5	21.5 ± 3.8	0.626	21.4 ± 2.9	21.7 ± 3.7	0.745
MNA-SF (mean ± SD)	9.7 ± 2.1	9.0 ± 3.0	0.074	9.5 ± 2.5	8.8 ± 2.9	0.110	8.9 ± 2.7	9.3 ± 2.7	0.524
Norton scale (mean ± SD)	10.1 ± 1.7	10.1 ± 2.4	0.797	10.3 ± 1.8	9.9 ± 2.5	0.215	9.2 ± 1.8	10.3 ± 2.1	0.024
Albumin (g/dL, mean ± SD)	3.7 ± 0.6	3.9 ± 0.6	0.033	3.7 ± 0.6	3.9 ± 0.6	0.204	3.8 ± 0.6	3.8 ± 0.6	0.746
Hb (g/dL, mean ± SD)	10.9 ± 1.4	11.1 ± 1.9	0.454	10.6 ± 1.4	11.6 ± 1.9	<.001	10.6 ± 1.5	11.1 ± 1.7	0.182
WBC (10^3^/μL, mean ± SD)	10.4 ± 3.3	10.5 ± 4.3	0.835	10.4 ± 4.2	10.4 ± 3.1	0.988	12.2 ± 7.3	10.2 ± 3.0	0.223

Abbreviations: *AHF* assisted hand feeding, *NGF* nasogastric tube feeding, *MNA-SF* Mini Nutritional Assessment–Short Form, *BMI* Body Mass Index

*p* < 0.05 for significant difference

**Table 3:** Risk factors of pneumonia, hospitalization and one-year mortality rates with logistic regression

	Pneumonia		Hospitalization		Mortality	
Factors	aOR	95% CI	aOR	95% CI	aOR	95% CI
Age	1.01	0.97–1.06	1.00	0.96–1.05	1.05	0.98–1.12
Sex (male)	1.96	0.97–3.97	1.64	0.74–3.62	0.44	0.13–1.42
Feeding (NGF)	2.20	0.92–5.30	1.94	0.82–4.60	2.38	0.58–9.70
Pressure sore	0.20	0.07–0.60	0.20	0.08–0.53	0.45	0.10–1.96
Barthel index	1.69	0.74–3.85	0.70	0.29–1.70	1.23	0.31–4.82
Albumin	0.58	0.32–1.03	–	–	–	–
Hb	–	–	0.63	0.50–0.80	–	–
Norton scale	–	–	–	–	0.76	0.59–0.98

Adjusted factors

Pneumonia: age, sex (male), feeding (NGF), pressure sore, Barthel index, albumin level

Hospitalization: age, sex (male), feeding (NGF), pressure sore, Barthel index, Hb

Mortality: age, sex (male), feeding (NGF), pressure sore, Barthel index, Norton scale

### Hospitalization rate and duration

Hospitalization rate was 54% in the AHF group and 66% in the NGF group (*p* = 0.162). Factors associated with the risk of hospitalization were absence of pressure sores (*p* = 0.002) and low Hb level (*p* < 0.001; Table 2). After adjusting for age, sex, feeding status, pressure sores, Barthel index and Hb level, pressure sores and high Hb were associated with protective factors (Table 3). Of note, mean hospitalization duration was 14.2 ± 26.4 and 17.8 ± 22.4 days in the AHF and NGF groups, respectively (*p* = 0.396). The frequency of hospital admission reveled no significant difference between AHF and NGF (*p* = 0.061).

### One-year mortality rate

One-year mortality rate was 8% in the AHF group and 15% in the NGF group (*p* = 0.415). We analyzed other factors to understand the association with one-year mortality rate. However, the results demonstrated only a significant difference in Norton scale (*p* = 0.024; Table 2). After adjusting for sex, age, feeding status, Barthel index, pressure sores, and Norton scale, the mortality rate was not significantly different between the NGF and AHF groups (aOR = 2.38; 95% CI, 0.58–9.70; Table 3).

## Discussion

The present study revealed that NGF is not associated with a decreased pneumonia risk in older patients with dementia who are cared for with in-home healthcare system. Meanwhile, hospitalization rate and one-year mortality were similar between AHF and NGF. However, all measured outcomes showed a similar, or rather less significant trend of increased risk in the NGF group. This study could provide preliminary evidence for tube feeding in home healthcare studies. Our study showed that the risk of pneumonia diagnosis was not significantly lower but revealed a nonsignificant trend of increasing risk of pneumonia in the NGF group. In older patients with dementia, gradual impairment of swallowing develops with disease progression. Owing to the loss of a normal clearance mechanism, the most frequent consequence is aspiration pneumonia, which typically occurs during eating when pharyngeal materials enter the lower airway, followed by infection with nonpathogenic flora [18].

A review of 19 cohorts suggested that tube feeding could not reduce the risk of aspiration pneumonia [19]. Subsequently, a study compared the incidence of aspiration pneumonia in individuals with NGF, gastrostomy, jejunostomy, and oral feeding. The authors found that after a 6-month follow-up, more patients (58%) in the tube feeding group developed aspiration pneumonia than in the oral feeding group (54% in NGF, 67% in the gastrostomy group, 75% in the jejunostomy group, and 17% in the oral feeding group). Furthermore, the study demonstrated a significant difference in risk of aspiration pneumonia between oral feeding and tube feeding (*p* < 0.01) [20]. Importantly, a recent study indicated that tube feeding was associated with around two-fold increased incidence of aspiration pneumonia diagnosis (RR = 2.32; 95% CI, 1.22–4.40) [21]. To be more specific, oral secretions and regurgitated gastric contents are severe causative factors for aspiration pneumonia, and feeding tubes provide no protection efficacy [19]. As opposed to stroke, dementia is a progressively deteriorating disease [22]. Individual strategies are required to manage different pathogeneses. Tube feeding is generally reserved for short-term (< 1 month), reversible, or unconscious individuals, such as those requiring post-surgery support and discharge. However, if the enteral tube feeding is anticipated more than 1 month, percutaneous endoscopic gastrostomy (PEG) is preferred because of less treatment failure, fewer complications and better nutritional status [23, 24] although one-year pneumonia risk showed no significant difference between the NGF group and the PEG group (aOR = 0.51; 95% CI, 0.18–1.47) [25]. For mild or moderate dementia, PEG placement should be preferred over the long- term placement of nasogastric tube, which was apparently the standard care in current guideline [26]. Of note, the initiation of tube feeding in patients with severe dementia is not recommended [26]. Additionally, pressure sores were potential predictive factors for pneumonia in the study participants. Proper positioning and regular position changing are useful strategies for the care of pressure sores [27]. Given the limited data available currently, position changing may be beneficial in pneumonia care [28].

With respect to hospitalization rate and duration, we found no significant difference in our study. A prior prospective observational study with 6-month follow-up also detected no difference in hospital admission between tube feeding and oral feeding [21]. Hospitalization leads to distress and is associated with poor outcomes in advanced dementia patients. However, older patients with dementia are frequent visitors to hospitals for several reasons. A prospective cohort study of 617 patients investigated the prevalence and mortality in an acute care setting revealed an increasing admission rate with age (29.6% for patients aged 70–79 years and 75% for patients aged > 90 years). Urinary tract infection or pneumonia was the primary cause. Moreover, these patients showed a higher mortality rate (adjusted mortality risk 4.02, 95% CI, 2.24–7.36) [29]. Most hospitalizations among older patients with dementia are avoidable. Additionally, invasive treatments in acute hospital care may not meet the needs of this group and lead to injuries. Even infectious diseases such as pneumonia could be treated effectively in a nursing home rather than in the hospital [30]. In the present study, the major cause of hospital admission was pneumonia. Increased Hb levels may be a protective factor for decreasing hospitalization rates and duration. Importantly, Hb could be an early indicator of the nutritional status of hospitalized older patients [31]. The home care system in Taiwan made great efforts to advocate the avoidance of frequent admissions and provided phone consultation as required [13].

The present retrospective observational study of home care residents showed no difference in mortality rate between AHF and NGF in patients with advanced dementia patients. However, this study revealed a nonsignificant trend of increased mortality rate in NGF group. A previous study suggested that 85.8% patients with advanced dementia (stage 7 on the Global Deterioration Scale) suffered from eating problems, with the 6-month mortality rate of 38.6% [32]. Furthermore, a longitudinal study in Taiwan investigated the survival outcome of patients with dementia. The authors concluded that 77% patients who were placed with a nasogastric tube died within 6 months. Therefore, nasogastric tube placement was recognized as a risk factor for increased 6-month mortality [33]. A 2009 Cochrane systemic review, which included seven observational studies, revealed insufficient evidence of increased survival rate in tube feeding groups [9]. A large prospective database study provided valuable evidence in survival analysis. They evaluated 36,429 nursing home residents with severe dementia and eating difficulty. In the study, 1956 residents were under PEG insertion compared with 34,536 residents who were fed orally. They found no significant difference of survival between both groups [34]. In line with earlier reports, our study did not favor tube feeding for decreasing the mortality rate. In previous studies, tube feeding group was mainly composed of PEG feeding. However, in this study, we added valuable evidence comparing AHG and NGF in mortality rate analysis. All above evidences may indicate that, once dementia progresses to a severe stage, with the development of eating problems, the mortality rate increases regardless of placement of feeding tube. In addition, increased one-year mortality rate was observed in subjects with low Norton scale in our study. Similar to prior studies, patients with lower Norton scale score were associated with higher mortality rate in older patients [35, 36].

Despite significant efforts to improve oral intake, most patients with dementia develop eating difficulties during the severe stage. A nasogastric tube was considered a convenient and efficient approach for individuals with feeding problems. However, NGF frequently presented with complications (e.g., blockage, dislodgement, pneumonia, trauma from insertion, and use of physical or chemical restraints) [37, 38]. When comparing NG and gastrostomy groups, a significantly higher number of tube-related complications were reported in the NG group (aOR = 0.18; 95% CI, 0.05–0.65) [25]. AHF in patients with advanced dementia should be considered an alternative to tube feeding [39]. Management of eating difficulties in advanced dementia remains controversial. The goals should emphasize on avoiding functional decline and adopting safe and effective oral feeding. A comprehensive swallowing assessment could help identify dysphagia etiology and offer adequate strategies for improving swallowing function. Furthermore, several approaches can increase the efficiency of careful hand feeding (e.g., modifying the feeding position, feeding skills, and changing both the feeding environment and texture of food). Of note, feeding skills are essential for preventing aspiration (e.g., the chin tuck posture and head rotation toward the weak side). Patients with an impaired oropharyngeal musculature can more easily control thickened food than thin fluids. Modifying the food texture of solids or liquids increases feeding effectiveness, particularly in patients with severe dementia [40]. Nevertheless, making the modified food acceptable and appetizing is vital to increase compliance. Furthermore, an adequate environment setting (e.g., removing distractions and scheduling mealtime with family) could make patients enjoy their social time, as opposed to isolated tube feeding. In the present study, well-trained nurses provided all these useful strategies for oral feeding of residents at the home health care system. To date, tube feeding still plays a role because of individuals’ concerns over the discomfort of being thirsty or hungry. However, the consequences of forgoing tube feeding (e.g., discomfort or pain) have not been investigated thus far. A prospective observational study of 178 nursing home patients attempted to address this issue. They observed different items creating a discomfort index. Shortness of breath, restlessness, observation of pain, and dehydration were associated with increased discomfort. Given the evidence that discomfort levels were not associated with forgoing tube feeding [41], additional efforts could be placed on evaluating the patients’ quality of life [42].

Our study expanded the study group from nursing home to home healthcare in the community settings, which is one of the significant care systems for severe dementia in Taiwan. Furthermore, relatively large number of patients receiving NGF is a useful comparison with other Asian countries, where the prevalence of NGF is high.

In hospitals or nursing homes, well-equipped facilities and specialized health care provide a high quality of care. Meanwhile, there was only one or no caregiver in the home care system, with inadequate training. Results from the home care system are essential for community practice. We believe that this preliminary study of tube feeding issues in subjects with severe dementia requiring home healthcare in Taiwan will help healthcare providers, patients, and families by providing a more realistic understanding of nutritional support in this population.

The present study has several limitations. First, for ethical reasons, conducting randomized controlled trials would be extremely challenging. In this retrospective study, the recruited subjects were already receiving NGF or AHF, and were not randomly enrolled. Therefore, the baseline characteristics of both groups were different. We restricted the study group only to complete bedridden status and severe dementia to make a balance in the study. Although we took the effort to select more comparable subjects in the NGF and AHF groups, the patients belonging to the NGF group were more compromised, as shown by the lower Barthel index and albumin level. Furthermore, intervention (NGF) and control (AHF) groups were not well matched, and the difference of sample size between these two groups was not negligible. However, this could also explain the high preference of NGF in patients with severe dementia because of its convenience and efficiency. Moreover, if outcome data from multiple centers could be collected, the study groups could be widely applied in general home healthcare population. Finally, no data were available on the following aspects: quality of life, objective assessment of discomfort, pain scale, use of physical and chemical restraints, and physical function. Quality of life is more important than life span, and it is usually neglected.

## Conclusions

The hallmarks of severe dementia are the lack of insight and capacity to make an independent decision. However, life-sustaining interventions have been widely applied, particularly with the use of feeding tube. Our study demonstrated that NGF was not associated with decreased risk of pneumonia compared with AHF and offered no benefits in terms of hospitalization and mortality in patients with severe dementia requiring home healthcare. Moreover, the burdens of NG tube placement could impair quality of life and lead to numerous complications. We believe that a dedicated attempt at AHF should be made in majority of patients in severe stages. Large-scale studies with prospective or randomized designs in home healthcare system are required to provide robust evidence.

## Data Availability

The datasets and analyzed during the current study is available from the corresponding author on reasonable request.

## Abbreviations

AHF: Assisted hand feeding
BMI: Body Mass Index
ECOG: Eastern Cooperative Oncology Group
FAST: Functional assessment staging
MNA: Mini Nutritional Assessment
MNA-SF: Mini Nutritional Assessment–Short Form
NGF: nasogastric tube feeding
